# HPV vaccination and sexual behaviour in healthcare seeking young women in Luxembourg

**DOI:** 10.7717/peerj.8516

**Published:** 2020-02-10

**Authors:** Camille Soudeyns, Niko Speybroeck, Marc Brisson, Joël Mossong, Ardashel Latsuzbaia

**Affiliations:** 1 Public Health Faculty, Université Catholique de Louvain, Brussels, Belgium; 2 Department of Social and Preventive Medicine, Université Laval, Quebec, Canada; 3 Epidemiology and Microbial Genomics, Laboratoire National de Santé, Dudelange, Luxembourg

**Keywords:** HPV vaccination, Luxembourg, Assortativity of sexual mixing, Sexually transmitted infection, Human papillomavirus, Sexial behaviour

## Abstract

**Introduction:**

Human papillomavirus (HPV) is the most common sexually transmitted infection (STI) worldwide. Despite recommendations for HPV vaccination of young women from health authorities, parental concerns were raised whether vaccination could induce unsafe sexual behaviour in young women. Therefore, the primary aim of this study was to investigate if HPV vaccination in healthcare seeking adult women in Luxembourg was associated with unsafe sexual behaviour.

**Methods:**

Seven hundred twenty-nine women (mean age = 22.5; range 18–43 years) were recruited either at Luxembourg family planning centres or at private gynaecology practices. All participants completed a questionnaire on vaccination status and sexual behaviour. Poisson and logistic regressions were used to study the association between sexual behaviour and vaccination status (*N* = 538). Both models were restricted to women younger than 26 years, since the first cohort being vaccinated would be 25 years old at the time of sampling. Assortativity of sexual mixing by age was also assessed for further transmission modelling for women <30 years reporting age of last/current sexual partner (*N* = 649). Women older than 29 years were excluded from the assortativity analysis due to restricted sample size.

**Results:**

In total, 386/538 (71.8%) of participants reported receiving HPV vaccine. Vaccination uptake significantly varied by nationality and was higher in Portuguese 112/142 (78.9%) and in Luxembourgish 224/313(71.6%) residents, and lower in residents of other nationalities 50/83 (60.2%) (*p* = 0.011). HPV vaccination was not associated with unsafe sexual behaviour such as shorter relationship duration with current or last sexual partner (odds ratio (OR) = 1.05, 95% CI [0.94–1.16]), younger age of sexual debut (OR = 1.00, 95% CI [0.88–1.14]), increased number of lifetime sexual partners (OR = 0.95, 95% CI [0.87–1.03), higher age difference with sexual partner (OR = 1.01, 95% CI [0.95–1.08]), condom use (OR = 0.97, 95% CI [0.60–1.56]), nor with other factors like smoking (OR = 0.73, 95% CI [0.47–1.15]) and nationality. HPV vaccination was only associated with younger age (OR = 0.84, 95% CI [0.75–0.94]). Relationship duration, age of sexual debut, age difference with sexual partner, smoking, age and non-Portuguese foreign nationality were predictors of number of lifetime sexual partners. Assortativity analysis revealed that young women chose sexual partners who were 2.3 years older on average.

**Conclusions:**

Our study found no association between unsafe sexual behaviour and HPV vaccination.

## Introduction

Human papillomavirus (HPV) is the most common sexually transmitted infection (STI) worldwide (World Health Organisation, 2019) infecting more than 80% of the sexually active population at least once during their lifetime (Chesson et al., 2014). HPV is responsible for more than 60,000 cases of cervical cancer annually in the European Union alone, leading to more than 30,000 deaths (De Martel et al., 2017).

Based on HPV carcinogenesis knowledge, several prophylactic vaccines were developed to protect against HPV infection and cervical cancer. In Luxembourg, the national HPV vaccination programme was introduced in 2008, offering three doses of bivalent (BV) or quadrivalent (QV) vaccines free of charge to 12–17 years old girls. In 2015, changes in national vaccination policy limited vaccination to two doses of BV vaccine targeting 12–13 years old girls. When girls reached the recommended age, their parents received an information letter from National Health Insurance advising them to get vaccinated. HPV vaccines were administered by private health professionals, mainly paediatricians (Latsuzbaia et al., 2018). In 2018, following an update of WHO recommendations, the policy changed again offering the nonavalent vaccine for 9–13 year old girls and boys (Superior Council of Infectious Diseases, 2019).

Despite recommendations from health authorities (World Health Organisation, 2017), uptake of HPV vaccine remains lower compared to other vaccines in many countries (Bruni et al., 2016). In Luxembourg, a previous study reported the national HPV vaccination coverage of 62%, ranging geographically from 38% to 79% with higher rates in the South–West and lower in the North and Centre of the country. The vaccination coverage significantly varied by nationality, with higher coverage rates in Portuguese and Former Yugoslavs and lower in Luxembourgish and other nationalities (Latsuzbaia et al., 2018). HPV vaccine uptake could be influenced by parental concerns regarding the vaccination of minors against STI (Marlow et al., 2009; Schuler et al., 2011). HPV vaccination might induce unsafe sexual behaviour in young adults because of their perception of protection against all STIs, rather than only HPV. Therefore, vaccinated young women might engage in unsafe sexual behaviour (e.g., unprotected sexual intercourse, earlier sexual debut or promiscuity) (Saslow et al., 2007; Walton, Orenstein & Pickering, 2015).

During the last decade, various research groups have investigated the impact of HPV vaccination on sexual behaviour, although results were inconsistent. While some studies reported no association between vaccination and sexual behaviour, such as sexual debut (Hansen et al., 2014; Ruiz-Sternberg & Pinzon-Rondon, 2014), number of lifetime sexual partners (Cummings et al., 2012; Forster et al., 2012) and condom use (Mather, McCaffery & Juraskova, 2012; Mattebo et al., 2014), others reported unsafe sexual behaviour in unvaccinated cohorts (Kasting et al., 2016).

Interestingly, women with older sexual partners—a phenomenon described as disassortative age mixing—are more likely to acquire STIs, including HPV (Kraut-Becher & Aral, 2006). This concept refers to the degree of self-similarity of sexual partner based on a particular characteristic. For instance, having a sexual partner similar in age represents an assortative sexual mixing (Malagon et al., 2017). Sexual mixing is an important component for model predictions of HPV transmission, prevalence and vaccination impact (Walker et al., 2012).

The primary aim of our study was to investigate whether HPV vaccination was associated with unsafe sexual behaviour in sexually active, healthcare seeking adult women. It is a secondary analysis of sexual behaviour data collected for a vaccine surveillance study conducted in Luxembourg between November 2015 and December 2017 (Latsuzbaia et al., 2019). Additionally, we assessed assortativity by age of sexual mixing in this study population.

## Materials & Methods

### Study design and data collection

In total, 729 healthcare seeking young women were recruited for this cross-sectional study with mean age of 22.5 years (age range: 18 to 43 years) either at Luxembourg family planning centres or at private gynaecology practices between November 2015 and December 2017. All participants signed informed consent forms (Supplement 1) and completed themselves a one-page paper questionnaire regarding their sexual behaviour and vaccination status during a medical appointment with a gynaecologist (Supplement 1). Socio demographic data collected via this questionnaire included: age, nationality, age of current sexual partner or last sexual partner (if there was no partner at the time of the study), number of lifetime sexual partners, condom use, age at first intercourse, smoking, self-reported vaccination status, vaccine type and date. Women did not receive any compensation for participation. Inclusion criteria were: (a) female sex; (b) at least 18 years of age; (c) sexually active (d) not pregnant.

### Statistical analysis

In order to ensure that all participants had the opportunity to be vaccinated, we restricted the analysis to women aged from 18 to 25 years, since the first cohort of women being vaccinated would be 25 years old at the time of the study sampling. Women who were unaware of their HPV vaccination status (9.1%) were excluded, leading to a final sample size of 538 participants. Comparisons between proportions and means were assessed using Pearson’s Chi^2^ test and Student’s *t* test, respectively. Condom use and smoking status were re-categorized as ‘’ever user” and ‘’never user”. Nationalities were categorized as “Luxembourgish”, “Portuguese” and “Others”, which included 23 more nationalities. Categories were chosen to represent the most frequent nationalities seen in Luxembourg. Relationship duration was defined as the duration of the relationship in years with the current or the last partner if there was no relationship at the time of the study. The number of sexual partners was defined as an aggregate measure of the total number of lifetime sexual partners. Age difference was calculated by subtracting the age of the participant at the time of the data collection from the reported age of the last/current sexual partner.

We studied the association between vaccination status and sexual behaviour using simple and multivariable logistic regression. Since the mean number of sexual partners was significantly different in vaccinated and unvaccinated women, we performed simple and multivariable Poisson regression analysis to further study factors affecting the number of sexual partners (Table 1).

**Table 1:** Characteristics of study population by vaccination status (*n* = 538).

	Vaccinated		Unvaccinated		Differences
	***n***	**Mean (SD)**	***n***	**Mean (SD)**	***p*-value**^a^
Age	386	21.1 (2.1)	152	21.9 (2.1)	<0.001
Age at first intercourse	381	16.6 (1.7)	148	16.5 (2.1)	0.792
Number of sex partner(s)	373	2.98 (2.4)	148	3.9 (3.2)	<0.001
Difference of age with last sex partner	375	2.2 (3.2)	148	2.3 (3.3)	0.719
Duration of Partnership	346	2.6 (2.1)	140	2.4 (2.3)	0.538
Nationalities % (*n*)					0.011
Luxembourgish	71.6 (224)		28.4 (89)		
Portuguese	78.9 (112)		21.1 (30)		
Others	60.2 (50)		39.8 (33)		
Condom use % (*n*)					0.898
Never	72.2 (96)		27.8 (37)		
Ever	71.6 (290)		28.4 (115)		
Smoking status % (n)					0.008
Never	75.7 (258)		24.3 (83)		
Ever	65.0 (128)		35.0 (69)		

**Notes.**

*n* number of participants % percentage SD Standard deviation

a Student’s *t*-test for quantitative variables, Chi^2^ test for categorical variables.

Variables considered at-risk in terms of sexual health were included in both models: number of sexual partners, condom use, duration of the relationship with the last/current partner in years, age difference between sex partners and age at first intercourse (Kasting et al., 2016). Cigarette smoking was included in both models, since previous studies have shown that smoking was associated with unsafe sexual behaviour (Hansen et al., 2010). Models were adjusted for nationality and age, since both variables affected vaccination uptake (Latsuzbaia et al., 2019; Latsuzbaia et al., 2018). Since the effect of an exposure on an outcome can be masked by intermediate and confounding variables (Westreich & Greenland, 2013), to estimate the total effects size of the variables of interests, we studied each sexual behaviour variable in separate models adjusting for smoking, nationality and age. Statistical analyses were performed using IBM SPSS Statistics25 (Armonk, New York) and STATA 14 (College Station, Texas).

For the second part of the study, we evaluated the assortativity of sexual mixing matrix by age. This type of analysis provides important information to understand how women in Luxembourg choose sexual partners with regard to age and how STIs, including HPV may transmit in this population. Since the purpose of this part was not related to vaccination status, we extended the sample to women aged between 18 and 30 years, leading to a sample size of 649 participants. We removed participants aged ≥ 30 years (13/729; 1.8%) since results would not have been representative for these age ranges due to the small sample size.

### Ethical approval

The study was approved by the Comité National d’Ethique de Recherche (CNER # 201501/02) and authorized by the Commission Nationale pour la Protection des Données (CNPD 288/2016).

## Results

### Demographic characteristics

**Figure 1:**
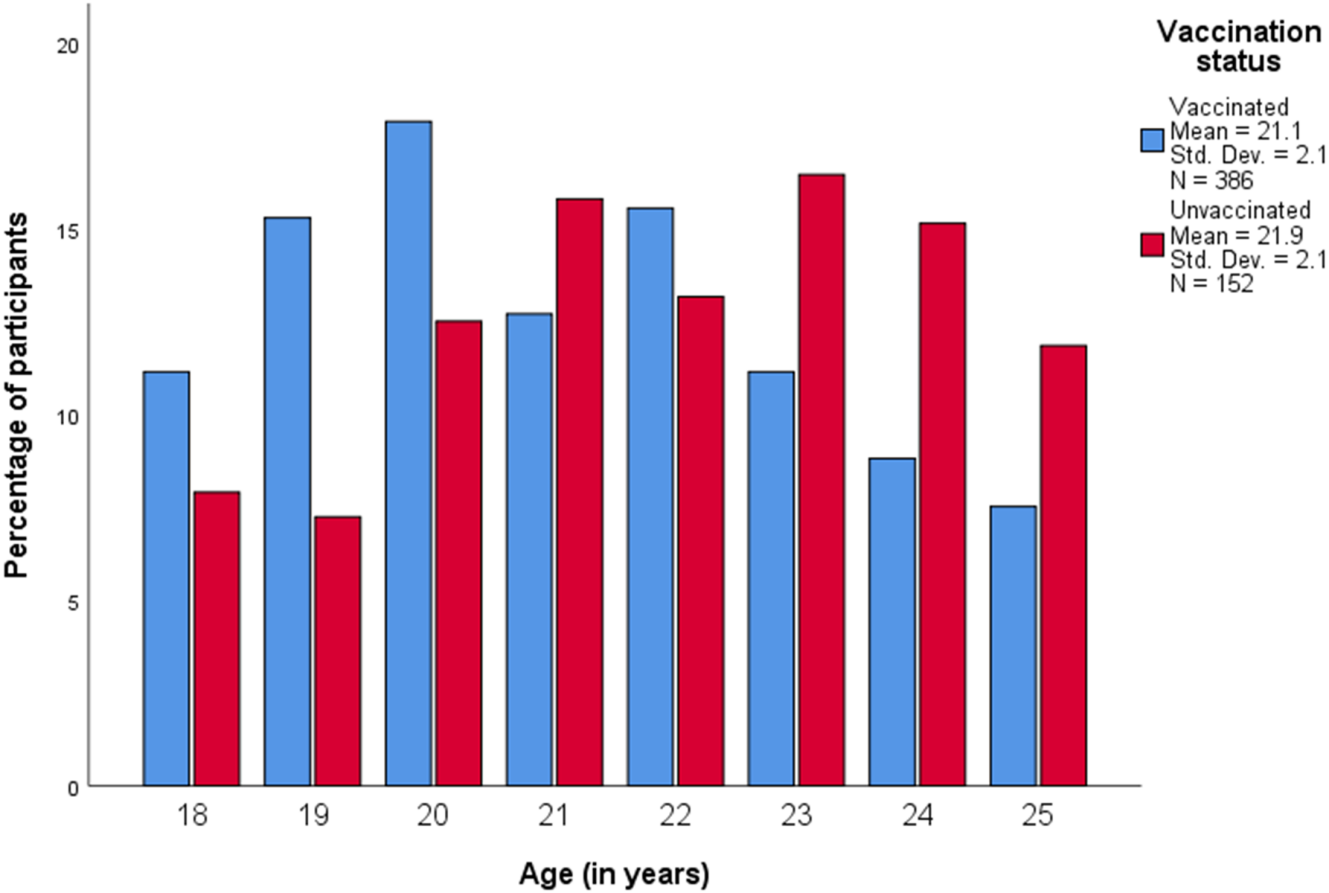
Age distribution of study participants according to HPV vaccination status. Std. Dev., Standard deviation; N, Number of participants.

Table 1 summarizes demographic and behavioural characteristics of 538 participants of whom 386/538 (71.8%) reported to be vaccinated. Figure 1 displays the age distribution of study participants by vaccination status (mean age was 21.3 years (standard deviation (SD) 2.1)).

**Figure 2:**
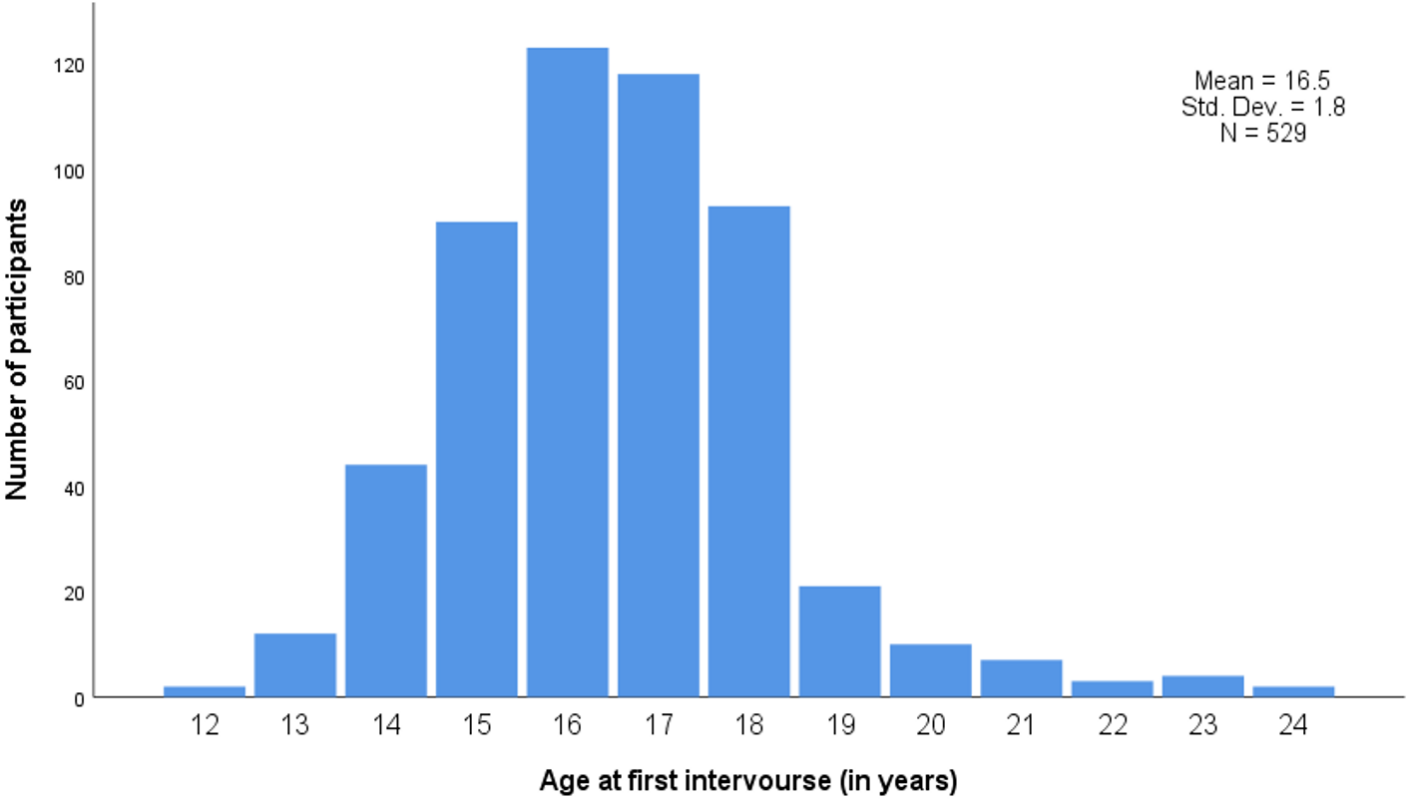
Age at first sexual intercourse. Std. Dev., Standard deviation; N, Number of participants.

Participants in the unvaccinated group were slightly older (mean age: 21.9 years) than participants in the vaccinated group (mean age: 21.1 years, *p* < 0.001) (Table 1). Vaccine uptake significantly varied by nationality and was higher in Portuguese 112/142 (78.9%) and in Luxembourgish 224/313 (71.6%) residents, and lower in residents of other nationalities 50/83 (60.2%) (*p* = 0.011). Mean age at first sexual intercourse was 16.5 years (Fig. 2), and did not differ according to HPV vaccination status (*p* = 0.792) (Table 1).

Last/current sexual relationship duration and condom use were not significantly different between groups (*p* = 0.538). Unvaccinated women reported a significantly higher number of sexual partners (3.89 sexual partners, SD = 3.27) compared to vaccinated women (2.98 sexual partners, SD = 2.43) (*p* < 0.001). Cigarette smoking was significantly higher in unvaccinated compared to vaccinated women (*p* = 0.008) (Table 1).

### Vaccination status and sexual behaviour

In simple logistic regression analysis, vaccination was associated with decreased lifetime number of sexual partners (odds ratio (OR) = 0.89, 95% CI [0.83–0.96]), younger age (OR = 0.84, 95% CI [0.75–0.94]), smoking (OR = 0.60, 95% CI [0.41–0.88]) and non-Portuguese foreign nationality (OR = 0.61, 95% CI [0.36–1.00]). In the adjusted analysis, only age remained significant, (OR = 0.84, 95% CI [0.75–0.94]) (Table 2). The total effect size for each exposure of interest is reported in Table 3.

**Table 2:** Simple and multivariable logistic regression analysis of factors associated with self-reported HPV vaccination.

	**Simple regression**			**Multivariable regression**		
Variable	OR	*P*-value	95% CI	OR	*P*-value	95% CI
Sexual behaviour						
Relationship duration (0–12 years)	1.03	0.537	[0.94–1.16]	1.05	0.380	[0.94–1.16]
Age at first intercourse (12–24 years)	1.01	0.791	[0.91–1.13]	1.00	0.974	[0.88–1.14]
Lifetime number of sexual partners (1–25)	0.89	0.001	[0.83–0.96]	0.95	0.214	[0.87–1.03]
Age difference with sexual partner (7–17)	1.01	0.718	[0.95–1.07]	1.01	0.809	[0.95–1.08]
Condom use						
Ever	Ref.			Ref.		
Never	0.97	0.898	[0.63–1.50]	0.97	0.893	[0.60–1.56]
Smoking status						
Never	Ref.			Ref.		
Ever	0.60	0.008	[0.41–0.88]	0.73	0.171	[0.47–1.15]
Age (18–25 years)	0.84	<0.001	[0.77–0.92]	0.84	0.002	[0.75–0.94]
Nationality						
Luxembourgish	Ref.			Ref.		
Portuguese	1.48	0.101	[0.92–2.37]	1.27	0.369	[0.76–2.12]
Others	0.61	0.048	[0.36–1.00]	0.78	0.397	[0.44–1.38]
Intercept	–	–	–	129.7	0.001	[8.15–2064.3]

**Notes.**

CI confidence interval Ref. reference OR Odds ratio

### Factors associated with numbers of sexual partners

In simple Poisson Regression analysis, HPV vaccination (rate ratio (RR) = 0.77, 95% CI [0.69–0.85]), longer relationship duration (RR = 0.93, 95% CI [0.91–0.96]), lower age at first intercourse (RR = 0.85, 95% CI [0.83–0.88]), lower age difference with sexual partner (RR = 0.97, 95% CI [0.96–0.98]) and Portuguese nationality (RR = 0.80, 95% CI [0.71–0.91]) were associated with lower number of lifetime sexual partners. Smoking (RR = 1.72, 95% CI [1.56–1.89]), higher age (RR = 1.10, 95% CI [1.07–1.12]) and non-Portuguese foreign nationality (RR = 1.40, 95% CI [1.24–1.58]) were associated with higher number of lifetime sexual partners. In multivariable regression, relationship duration, age at first intercourse, age difference with sexual partner, smoking, age and non-Portuguese foreign nationality remained significant, generally with slightly weaker effects (Table 4). The total effect size for each exposure of interest is reported in Table 5.

**Table 3:** Multivariable logistic regression analysis of factors associated with self-reported HPV vaccination: effect size of exposure variables.

Models	Variable	OR	*P*-value	95% CI
	Sexual behaviour			
Model 1^a^	Relationship duration	1.05	0.294	[0.96–1.16]
Model 2^a^	Age at first intercourse	1.02	0.760	[0.91–1.14]
Model 3^a^	Lifetime number of sexual partners	0.94	0.128	[0.88–1.02]
Model 4^a^	Age difference with sexual partner	1.01	0.808	[0.95–1.07]
Model 5^a^	Condom use			
	Ever	ref		
	Never	0.89	0.626	[0.57–1.40]
Model 6^b^	Smoking status			
	Never	ref		
	Ever	0.62	0.018	[0.42–0.92]

**Notes.**

CI confidence interval Ref. reference OR Odds ratio

a Adjusted for age, smoking and nationality.

b Adjusted for age and nationality.

### Sexual mixing assortativity

**Figure 3:**
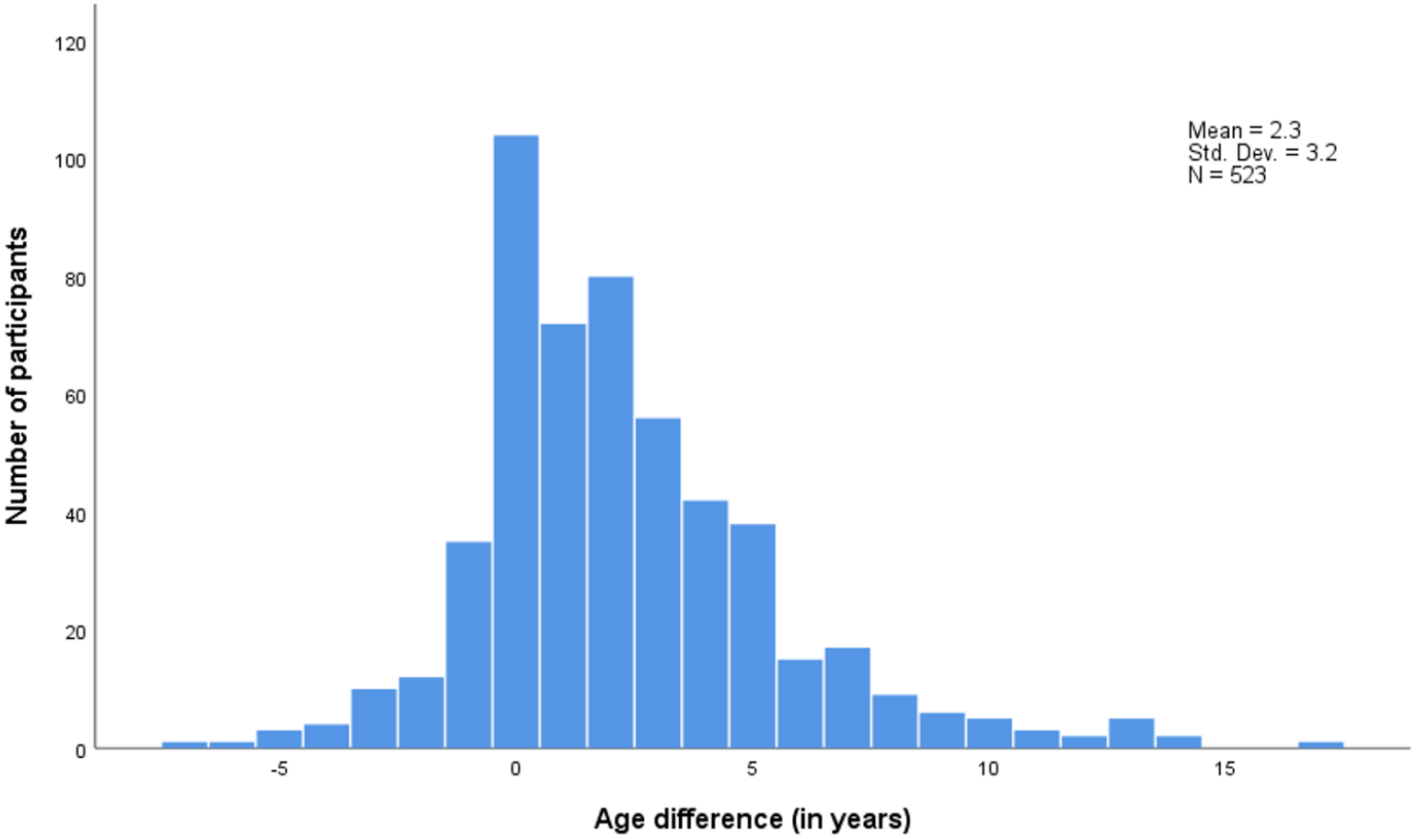
Age difference between sexual partners (Sexual partner’s age –participant’s age). Std. Dev., Standard deviation–N, Number of participants.

Figure 3 shows that the distribution of partner age differences is slightly skewed to the right with women’s partners being 2.3 year older than themselves on average.

**Figure 4:**
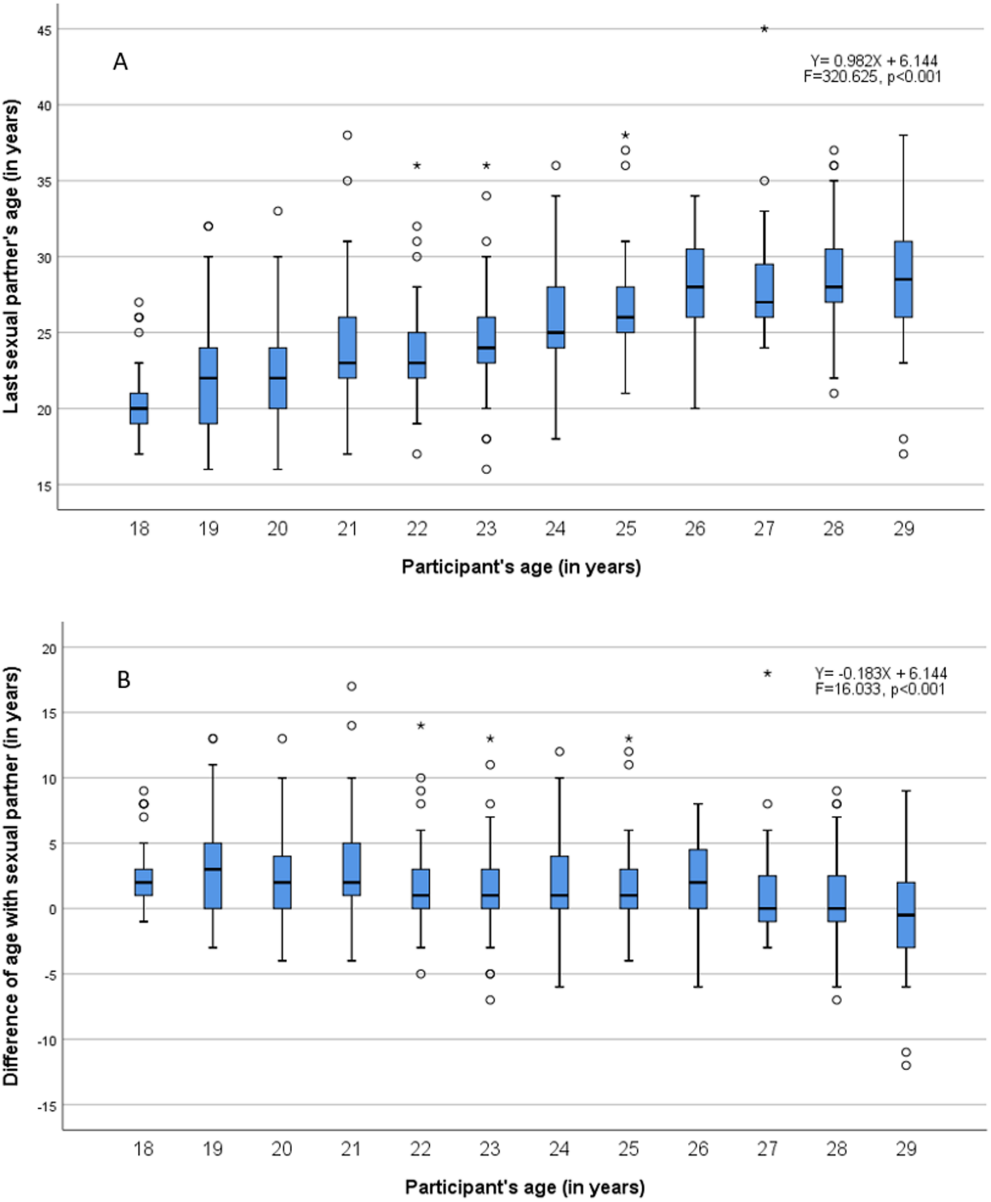
Sexual mixing by age of sexual partner (A) and by age difference with sexual partner (B).

Figure 4A shows a significant linear increase of last/current sexual partner’s age with participant age (*p* < 0.001). Figure 4B shows a significant linear decrease of age difference with the increase of participants age (*p* < 0.001), i.e., the age difference in sexual mixing tends to decrease with increasing age.

**Table 4:** Simple and multivariable Poisson regression analysis of lifetime number of sexual partners.

	**Simple regression**			**Multivariable regression**		
Variable	RR	*P*-value	95% CI	RR	*P*-value	95% CI
Vaccinated	0.77	<0.001	[0.69–0.85]	0.95	0.382	[0.85–1.06]
Sexual behaviour						
Relationship duration (0–12 years)	0.93	<0.001	[0.91–0.96]	0.92	<0.001	[0.90–0.94]
Age at first intercourse (12–24 years)	0.85	<0.001	[0.83–0.88]	0.86	<0.001	[0.83–0.88]
Age difference with sexual partner (−17–7 years)	0.97	<0.001	[0.96–0.98]	0.98	0.016	[0.97–1.00]
Condoms use						
Never	Ref.			Ref.		
Ever	0.96	0.665	[0.92–1.14]	1.02	0.619	[0.92–1.16]
Smoking status						
Never	Ref.			Ref.		
Ever	1.72	<0.001	[1.56–1.89]	1.41	<0.001	[1.27–1.56]
Age (18–25 years)	1.10	<0.001	[1.07–1.12]	1.12	<0.001	[1.10–1.15]
Nationality						
Luxembourgish	Ref.			Ref.		
Portuguese	0.80	<0.001	[0.71–0.91]	0.88	0.051	[0.77–1.00]
Others	1.40	<0.001	[1.24;1.58]	1.24	0.001	[1.09;1.41]
Intercept	–	–	–	3.29	0.001	[1.60–6.75]

**Notes.**

CI confidence interval Ref. reference RR rate ratio

## Discussion

This is the first study in Luxembourg to evaluate the association of HPV vaccination with sexual behaviour. Our results show no association between HPV vaccination and unsafe sexual behavior in adult women.

Our study thus adds further evidence to a number of studies reporting a lack of association between HPV vaccination and sexual behaviour, such as number of lifetime sexual partners or age at sexual debut (Kasting et al., 2016; Rysavy et al., 2014; Sadler et al., 2015). In a large cross-sectional survey from Denmark of 40,000 women including 3,800 HPV vaccine recipients, no effect of vaccination on age of sexual debut or number of lifetime sexual partners was observed, although unvaccinated women were less likely to use contraceptives during sexual debut (Hansen et al., 2014).

In a large longitudinal study based on insurance data of more than 200,000 participants, HPV vaccination was associated with higher STI rates, which is a reliable indicator of unsafe sexual behaviour (Jena, Goldman & Seabury, 2015). Since STI rates increased also in unvaccinated population during the same time-period, researchers concluded that HPV vaccination did not increase rates of STIs (Jena, Goldman & Seabury, 2015).

**Table 5:** Multivariable Poisson regression analysis of lifetime number of sexual partners: effect size of exposure variables.

Model	Variable	RR	*P*-value	95%CI
Model 1^a^	Vaccinated	0.85	0.002	[0.85–1.06]
	Sexual behaviour			
Model 2^b^	Relationship duration	0.94	<0.001	[0.91–0.95]
Model 3^b^	Age at first intercourse	0.86	<0.001	[0.84–0.89]
Model 4^b^	Age difference with sexual partner	0.98	0.001	[0.96–0.99]
Model 5^b^	Condoms use			
	Never	ref		
	Ever	1.09	0.124	[0.98–1.22]
Model 6^c^	Smoking status			
	Never	ref		
	Ever	1.66	<0.001	[1.51–1.83]

**Notes.**

CI confidence interval Ref. reference RR rate ratio

Adjusted for nationality and age.

a Adjusted for age, smoking and nationality.

b Adjusted for age and nationality.

In simple and multivariable models adjusted by age and nationality, the number of sexual partners was significantly lower in vaccinated women. However, this association was non-significant in the full model, suggesting the presence of interactions between the variables. Sadler et al. reported that unvaccinated young women were significantly more likely to have three or more sexual partner in the last 6 months compared to vaccinated women (Sadler et al., 2015). In our multivariable analysis, longer relationship duration, lower age at first intercourse, and lower partner age difference were associated with lower number of lifetime sexual partners. Smoking, higher age and non-Portuguese foreign nationality were associated with higher number of lifetime sexual partners. In a recent study in college-aged men and women in the US, alcohol use and race were independent predictors of sexual behaviour, whereas exposure of cigarette smoking was not collected (Brouwer et al., 2019).

Younger women tended to choose sexual partner who were on average 2 years older than themselves. Our results show that assortativity of age mixing increases with age, suggesting that older women preferred same-aged sexual partners. These results are similar to a survey conducted in England, where the median age difference between sexual partners was 2 years (Prah et al., 2015; Prah et al., 2015). Similarly to our study, the age difference was decreasing with older age: women aged between 16–24 years chose 3 years older sexual partners on average. Together these findings suggest that younger women are more prone to engage in dissortative age mixing, a behaviour likely to increase their risk for STIs.

Our study has several limitations. First, it is a secondary analysis of a wider vaccination surveillance project in Luxembourg, so the study was not primarily designed for this purpose. Second, the recruitment process was mainly performed at family planning centres in Luxembourg, where women seek sexual health services, thus our results may not be representative of the general population. The vaccination coverage in our study population (72%) was slightly higher than the national vaccination coverage estimates of 62% based on social security data (Latsuzbaia et al., 2018), although it should be noted that the age distribution of the cohorts are not the same.

We did not collect data on hormonal contraception use (emergency or counselling), other STIs screening, or sexual practice known to be enhancing risks for sexual health (anal intercourse, multiple sexual partnership, or group sex). Mattebo et al. found no significant association between condom use, STIs, and experiences of oral and anal sex, or friends-with-benefits relationships and HPV vaccination although vaccinated women were more likely to experience “one-night stands” (Mattebo et al., 2014).

Assortativity of sexual mixing by age has become useful for HPV transmission and HPV vaccination impact modelling (Choi et al., 2018). A strength of our study is that the data were collected in a medical context in the presence of gynaecologists making it less conducive to social desirability bias, observed when sexual behaviour is collected in school settings.

In further research, our age mixing data could be useful to study HPV transmission modelling and predicting the impact of HPV vaccination. Since young men have been recently integrated in HPV vaccination programs, it would be interesting to study effect of HPV vaccination on sexual behaviour in men. So far, the best strategies to help health care professionals to address vaccination hesitancy are dialogue-based interventions tailored to specific populations and their specific concerns. Yet there is a crucial need for new evidence-based strategies to face up to this major public health problem, which is not limited to HPV (Jarrett et al., 2015).

## Conclusions

Our study found no evidence of HPV vaccination influencing sexual behaviour.

##  Supplemental Information

10.7717/peerj.8516/supp-1 Supplemental Information 1 Questionnaire

10.7717/peerj.8516/supp-2 Supplemental Information 2 Papillux study dataset

## Additional Information and Declarations

### Data Availability

The following information was supplied regarding data availability: The raw data is available in the Supplemental Files.
