# Supplementary material for: HPV vaccination and sexual behaviour in healthcare seeking young women in Luxembourg
Source: PeerJ. 2020 Feb 10;8:e8516. doi: 10.7717/peerj.8516 (PMC7017793; doi:10.7717/peerj.8516)
Supplement: Supplemental Information 1 [file peerj-08-8516-s001.pdf]

Questionnaire

1. Have you been vaccinated against human papillomavirus (HPV) ?

yes ☐ no ☐ don't know ☐

If yes : date of 1st dose :___/ ___/ 20___ number of doses : ____

Vaccine : Gardasil ☐ Cervarix ☐ don't know ☐

2. Have you had sexual relations?

Yes ☐ No ☐

If yes :

a) How old were you at 1st sexual relationship: ____

b) How many partners have you had in total? (Please indicate all sexual partners, short or long duration).

Number of sexual partners: _____

c) Please indicate age and sex of your last sexual partner, as well as the calendar year when sexual relationship started:

Last partner age: ___ ; sex: M / F

Year of 1st sexual contact with partner: _____

3. Do you smoke cigarettes ?

Daily ☐ often ☐ sometimes ☐ never ☐

4. Do you use condoms ?

Always ☐ often ☐ sometimes ☐ never ☐
